# Updates on the Role of DXA in the Evaluation and Monitoring of Osteoporosis

**DOI:** 10.1007/s11926-025-01205-9

**Published:** 2025-11-01

**Authors:** Shahane Anupama, Sian Yik Lim, Marcy B. Bolster

**Affiliations:** 1 Division of Rheumatology, University of Pennsylvania, Philadelphia, PA USA; 2 Hawaii Pacific Health Medical Group, Honolulu, HI USA; 3 John E Burns School of Medicine University of Hawaii, Honolulu, US; 4 Division of Rheumatology, Allergy, and Immunology, Massachusetts General Hospital , Boston, US; 5 Harvard Medical School, Boston, MA USA

**Keywords:** Dual x-ray absorptiometry (DXA), Bone mineral density (BMD), FRAX, Fragility fracture, Risk assessment

## Abstract

**Purpose of review:**

Osteoporosis, the most common metabolic bone disease and a global health problem, is the result of abnormal bone architecture and bone loss, leading to bone fragility and an increased risk of fractures. A fragility or low-trauma fracture is the first clinical sign of osteoporosis and is typically associated with a significant risk of future fractures. Practical screening tools and guidelines are critical for fracture prevention. Dual X-ray absorptiometry (DXA) is the gold standard for risk stratification and diagnosis of osteoporosis. In this review, we report recent advances in in DXA screening and bone density evaluation for patients at risk of fragility fracture.

**Recent Findings:**

DXA measures bone density as an absolute measure (g/cm2) and also reports the patient’s bone mineral density (BMD) as a comparison to a standard population, which is the basis of the World Health Organization (WHO) classification for osteoporosis. DXA also provides vertebral fracture assessment (VFA), an additional tool to assess vertebral fracture presence and increased fracture risk. Understanding the methodology, reporting, and limitations of DXA use is critical for appropriate utilization of the test. BMD is validated as a predictor of fracture risk and may be used with or without clinical risk factors as part of the FRAX fracture risk assessment tool to accurately assess an individual’s fracture risk. In this review, we discuss the role of DXA in screening, diagnosis, and treatment of osteoporosis. We include FRAX and its limitations, trabecular bone score (TBS), quantitative computed tomography (qCT) the American College of Rheumatology (ACR) updates on the management of glucocorticoid-induced osteoporosis, the updated International Society of Clinical Densitometry (ISCD) Position Statements, and report the key features of DXA testing, reporting, and utilization in clinical practice.

**Summary:**

DXA plays a critical role in BMD evaluation and fracture risk assessment. Appropriate utilization of this tool in clinical practice, through accurate interpretation and application of results, is essential for effective fracture prevention.

## Introduction

Osteoporosis, a common metabolic bone disease, results from bone loss and abnormal bone architecture over time, with a consequent increase in fracture risk. Osteoporosis is a subclinical condition, lacking evident symptoms before the occurrence of a fracture. Effective screening tools and strategies are crucial for identifying individuals at high fracture risk and guiding the initiation of therapy. Dual-energy X-ray absorptiometry (DXA) is the most common test for screening and diagnosing osteoporosis. Here, we review the role of DXA in screening, diagnosis, and treatment of osteoporosis.

## Epidemiology

Osteoporosis is a national and global public health problem. It is projected that 12.3 million Americans are diagnosed with osteoporosis, with 2 million fragility fractures per year [1, 2]. Among White adults, 1 in 2 postmenopausal women and 1 in 5 men >50 years will sustain a fragility fracture in their lifetimes [3]. With an aging population, the incidence and prevalence of osteoporosis are expected to increase substantially. Annual fracture incidence is expected to increase to 3.2 million by 2040 [4]. Clinically, osteoporosis presents as fragility or insufficiency fracture(s), defined as those sustained with minimal to no trauma. The most common fragility fractures include vertebrae (lumbar spine), proximal femur (hip), and distal forearm (wrist).

### Societal Impact

Fragility fractures impose a significant socioeconomic burden on society related to high morbidity, fracture-related costs, and health care utilization. Hip fractures are associated with significant one-year mortality and are highly predictive of further fractures. An estimated 20% of patients with a hip fragility fracture require nursing home care [5]. Vertebral fractures are associated with considerable pain and disability as well as a 5-fold increase in risk of subsequent vertebral fractures. Wrist fractures, associated with a negative impact on quality of life, are seen more commonly in younger postmenopausal women, and these individuals are often not evaluated for underlying osteoporosis following this fragility fracture [6]. Fractures also impose a significant economic burden, resulting in increased hospitalizations, medical visits, and nursing home admissions. Annual fracture-related costs are estimated at $17.9 billion in the United States (US) and are expected to rise to over $95 billion worldwide by 2040 [4, 7].

## Osteoporosis Screening

Since osteoporosis is often asymptomatic, screening tools to detect low bone mass are crucial for identifying individuals at risk of fracture. DXA is the most commonly used method to measure bone mass or bone mineral density (BMD). Bone mineral density (BMD) is derived by dividing the amount of mineral content scanned by the area measured [8]. It is reported in absolute terms as grams of mineral per square centimeter scanned (g/cm2) and in relative terms in comparison to a defined population. Bone density measured at a specific site best correlates with fracture risk at that site but is also predictive of fracture risk at other sites [9]. Central or axial DXA measures BMD at the lumbar spine, total hip, and femoral neck. BMD measurement at the hip is considered most accurate [9] and reflective of the risk of hip fracture. BMD measurement at two sites is recommended in all patients, and the most predictive sites include the lumbar spine and hip [10]. Peripheral DXA measures the radius, which is recommended when two sites are otherwise not feasible, such as in patients with bilateral hip arthroplasty, prior spine surgery, or those with advanced lumbar degenerative disease. The radius is also recommended for DXA in conditions specifically affecting forearm BMD, such as hyperparathyroidism [11].

## DXA Interpretation

DXA is the preferred method for assessing bone density and is considered the gold standard for diagnosing osteoporosis. The diagnosis of osteoporosis is widely based on the World Health Organization (WHO) criteria established in 1994 [12], which utilize T-scores to define normal bone mass, low bone mass, and osteoporosis. Each patient’s BMD is compared to a standard population – an age-, sex-, and ethnicity-matched reference population (Z-score) or a young adult reference population (T-score). The International Society of Clinical Densitometry (ISCD) recommends a white (non-race/ethnicity adjusted) young female normative database for all populations, irrespective of race and gender [10]. T-scores and Z-scores are calculated by obtaining the difference between the patient’s BMD and that of the reference population and dividing the difference by the standard deviation (SD) for the reference population [13]. A Z-score of − 2.0 or lower in premenopausal women indicates low bone mass for age and should prompt an evaluation for secondary causes of bone loss. The reference standard for T-score is the female, white, age 20–29, NHANES II database. For individuals over 50 years old, low bone mass is defined as a T-score between − 1.0 and − 2.5, and osteoporosis is defined as a T-score of ≤−2.5 (Table 1). Pharmacologic therapy for fracture prevention is recommended in patients with a T-score ≤ −2.5, irrespective of their prior fracture history.

**Table 1:** World health organization (WHO) definition of osteoporosis and osteopenia

Normal	T-score >−1.0
Low bone mass	T-score − 1.0 to −2.5
Osteoporosis	T-score ≤−2.5

While this classification helps identify populations at risk for fracture based on BMD, several studies have shown that most women presenting with fragility fractures have a BMD higher than 2.5 SD below the mean and would thus not be classified as having osteoporosis based on these criteria alone [14–16]. Several factors contribute to an increased risk of fractures, including abnormal bone architecture, bone loss, and a higher likelihood of falls. There are modifiable and non-modifiable risk factors contributing to fracture risk, including age, parental history of hip fracture, certain medications such as glucocorticoids or aromatase inhibitors, and chronic medical conditions. The Fracture Risk Assessment Tool (FRAX) is a validated tool that utilizes several risk factors to predict the 10-year probability of hip and major osteoporotic fracture, providing a framework to distinguish those patients with osteopenia who may be at high fracture risk.

## FRAX

FRAX, developed by the University of Sheffield [17], is a widely used, readily available fracture risk assessment tool [17]. It incorporates the femoral neck BMD and clinical risk factors to assess fracture risk [17]. FRAX was developed based on primary data from 9 population-based cohorts derived from North America, Europe, Asia, and Australia. FRAX was subsequently validated in 11 independent cohorts with a similar geographic distribution [18]. Several clinical risk factors were identified through meta-analyses of these population-based studies [19]. These factors are weighted differently in the FRAX calculation, as each factor affects fracture risk to a varying degree. Importantly, the FRAX tool is also validated for use without BMD information; thus, in areas without easy access to DXA cans, a fracture risk assessment can be calculated based solely on risk factors [17].

FRAX integrates clinical risk factors with femoral neck BMD to calculate the 10-year probability of a major osteoporotic fracture and hip fracture, enabling more accurate risk assessment in patients with low bone mass. Risk factors included in FRAX are listed in Table 2. FRAX has been evaluated by multiple studies and is integrated into several osteoporosis guidelines worldwide [20].

**Table 2:** FRAX risk factors –

Age	Prior osteoporotic fracture
Sex	Rheumatoid arthritis
Parental history of hip fracture	Alcohol intake (> 3 units/day)
Oral glucocorticoids	Smoking (current)
Body Mass Index	Bone Mineral Density at femoral neck
Secondary causes of osteoporosis (type I (insulin dependent) diabetes, osteogenesis imperfecta in adults, untreated long-standing hyperthyroidism, hypogonadism or premature menopause (< 45 years), chronic malnutrition or malabsorption and chronic liver disease)	

FRAX is validated for use in men and women aged 40–90 years and was tested in treatment-naïve patients. While not indicated for patients currently on therapy, it may be used to assess fracture risk in patients who have been off bisphosphonate therapy for at least 2 years, or off non-bisphosphonate treatment for 1 year [21, 22]. The FRAX score provides a quantitative estimate of fracture risk and is particularly helpful in guiding therapy in patients with osteopenia. A significant proportion of postmenopausal women sustain a hip fracture with a T-score greater than − 2.5 [23]. In a study of 149,524 White postmenopausal women, while fracture rates in patients with a T-score of −2.5 or less were the highest, they experienced only 18% of the osteoporotic fractures, and 26% of the hip fractures. While T score ≤−2.5 is associated with high fracture risk, majority of fragility fractures are seen in patients with T scores higher than − 2.5 [24].

An important limitation of FRAX is that the clinical risk factors are binary inputs, which do not account for dose-response relationships. For example, the number of prior fractures, glucocorticoid dose and duration, and amount of alcohol consumed have a dose-response relationship to fracture risk. Furthermore, many factors that contribute to fracture risk are not included in the FRAX calculation, including other relevant details such as the recency of fractures, history of falls, medical conditions associated with fracture risk (such as diabetes mellitus and sarcopenia), and certain medications (aromatase inhibitors, proton pump inhibitors, and seizure medications). Similarly, the lumbar spine bone mineral density is not included in the FRAX calculation.

FRAX is country-specific, as fracture epidemiology and mortality rates vary among different racial and ethnic groups, and fracture rates exhibit global variability. Therefore, FRAX models need to be calibrated to specific countries based on unique epidemiological data of fractures and fracture-associated mortality [18]. To address several limitations of the FRAX tool, FRAXplus incorporates fall history [25], high steroid dose (>7.5 mg daily) [26], fracture recency [27], concurrent data on lumbar spine BMD [28], diabetes duration [29], trabecular bone score (TBS) [30] and hip axis length, providing a better assessment of fracture risk.

## Glucocorticoid-induced Osteoporosis Updated Guidelines 2022

The updated American College of Rheumatology guidelines for the prevention and treatment of glucocorticoid-induced osteoporosis (GIOP) are applicable to patients on either high-dose or long-term glucocorticoid (GC) therapy [31]. Significant bone loss has been shown to occur within the first 3 to 6 months of glucocorticoid therapy [32], and risk stratification is recommended for all patients on prednisone equivalents of 2.5 mg or more per day for 3 months or longer. Initial fracture risk assessment including FRAX (age >40 years) and BMD assessment with VFA or spine X-ray is recommended for all patients initiating GC therapy. Risk assessment is categorized as low, moderate, high, and very high risk based on FRAX scores as well as GC dosing (Table 3). For patients taking GC 2.5 mg or more but less than 7.5 mg daily who are considered low risk and do not initiate pharmacotherapy, a repeat assessment is recommended after 1–2 years. Repeat risk assessments every 1–2 years are recommended for all patients with moderate, high, and very high fracture risk.

**Table 3:** Glucocorticoid induced osteoporosis risk Stratification (Adapted from American college of rheumatology Glucocorticoid-Induced osteoporosis guidelines 2022)

Risk	Definition
Low risk	GC dose < 7.5 mg/day AND T score >−2.0 OR Z score >−3.0 AND No additional clinical risk factors
Moderate risk (< 40 years)	≥ 7.5 mg/day for ≥ 6 months AND Z-score ≤ −3.0 OR Significant bone loss over 1–2 years
Moderate risk (> 40 years)	FRAX 10-year MOF risk 10–20% OR Hip fracture risk 1–3% OR T-score between − 1.0 and − 2.4
High risk	T score ≤ 2.5 and >−3.5 OR FRAX 10-year MOF risk 20–30% OR Hip fracture risk 3–4.5.5%
Very high risk	Prior OP fracture(s) OR T-score ≤ 3.5 OR FRAX 10-year MOF risk ≥ 30% OR Hip fracture risk ≥ 4.5% OR High GC ≥ 30 mg/day for > 30 days OR Cumulative GC dose ≥ 5 g/year

Fracture risk assessment for patients on long-term or high dose GC therapy

*MOF* major osteoporotic fracture

## Vertebral Fracture Assessment (VFA)

Vertebral insufficiency fractures are the most common osteoporotic fractures, typically seen in adults over 50 years. Vertebral fractures are associated with significant morbidity, as well as a 5-fold increase in risk of further vertebral fractures and a 2–3 fold increase in other osteoporotic fractures [33]. Approximately two-thirds of vertebral fractures are asymptomatic and hence not clinically recognized, but rather may be found incidentally on chest x-rays or chest and abdomen CT scans. Furthermore, studies have shown that a large number of women with asymptomatic vertebral fractures may have a T score ≥−2.5. Irrespective of BMD, the presence of a vertebral fracture is diagnostic of osteoporosis and anti-fracture therapy is recommended to decrease risk of further fracture [34].

DXA-assisted vertebral fracture assessment (DXA-VFA) is an effective method for screening for vertebral fractures [35]. It is a convenient, low-cost, and low-radiation alternative to traditional X-rays of the lateral thoracic and lumbar spine. It is performed during BMD measurement using DXA technology [35]. Compared to standard radiographs, however, DXA-VFA may provide an inferior image with poor visualization of the spine above the T7 level. Improvements in DXA technology, with higher resolution of newer scanners, have improved the detection of mild vertebral fractures [35]. Interpreter variability and experience may affect the validity of DXA-VFA reports, and this can be reduced by automating VFA and improving observer training and inter-observer agreement [36].

In general, DXA-VFA or traditional imaging is recommended in individuals at high risk of vertebral fractures, such as progressive height loss >1.5 inches, patients receiving glucocorticoid therapy, and patients with a history of fragility fractures [37–39]. In patients with continued high vertebral fracture risk, DXA-VFA should be repeated every 2–3 years as clinically warranted [40].

## Treatment Recommendations

The Bone Health and Osteoporosis Foundation (BHOF), formerly the National Osteoporosis Foundation (NOF), guidelines recommend initiating anti-fracture therapy based on DXA and FRAX thresholds as determined by population-based studies and a cost-benefit analysis [41]. Primary fracture prevention, treatment goal for preventing the first fracture, is recommended for patients with a DXA scan revealing a T-score ≤−2.5 at the lumbar spine, total hip, or femoral neck, and for patients with low bone mass (T-core − 1.0 to − 2.5) with ≥ 20% predicted10-year probability of major osteoporotic fracture (hip, spine, humerus, forearm) or ≥ 3% predicted10-year probability of hip fracture (Table 4).

**Table 4:** BHOF indications for osteoporosis treatment. Primary and secondary fracture prevention

Primary fracture prevention DXA with T score ≤−2.5 at femoral neck, total hip, lumbar spine. DXA with T score − 1.0 to −2.5 and FRAX scores greater than or equal to 20% for major osteoporotic fracture or 3% for hip fracture.
Secondary fracture prevention Insufficiency fracture of the hip or vertebra, regardless of BMD. Insufficiency fracture of the distal radius, proximal humerus, or pelvis in individuals with low bone mass (T score between − 1.0 and − 2.5)

A previous fragility fracture is one of the strongest predictors of future osteoporotic fractures. Secondary fracture prevention aims to reduce the risk of further fractures after the sentinel fracture. Low-trauma hip and vertebral fractures are indications for initiation of therapy irrespective of BMD. Similarly, treatment is indicated in patients with low bone mass who sustain a low-trauma fracture of the proximal humerus, distal radius, or pelvis.

## DXA guidelines – screening

Bone density screening is recommended for individuals at risk of fracture, where testing is expected to inform clinical care (Table 4). Individual decisions should be made based on a clinical risk profile that combines age and risk factors for bone loss. The BHOF recommends screening in women 65 years and older and men 70 years and older, as well as in women aged 50–64 years and men aged 50–69 years who have osteoporosis risk factors [13]. DXA is also recommended in women and men with prior fragility fracture(s). In premenopausal women and younger men, screening densitometry should be considered in those with a significant history of fragility fractures or ongoing significant risks for bone loss; however, DXA is not recommended for routine screening in these populations. Table 5 summarizes screening densitometry recommendations from BHOF, which align with Endocrine Society guidelines and include the 2023 updated recommendations from ISCD [10, 13, 42, 43].

**Table 5:** Screening guidelines

DXA – Women > 65 years and men > 70 years regardless of clinical factors Postmenopausal women < 65 years, women in menopause transition, and men < 70 years with clinical risk factors - Low body weight - Prior fracture - High risk medication use - Medical conditions associated with bone loss Adults with a fragility fracture history Adults with a disease or condition associated with low bone mass or bone loss Adults taking medications associated with low bone mass or bone loss. Anyone being considered for pharmacologic therapy Anyone being treated should be monitored to monitor treatment effect
VFA – T score <−1.0 and - Women > 70 years or men > 80 years - Historical height loss > 4 cm (1.5 inches) - Self reported but undocumented prior vertebral fracture - Glucocorticoid therapy equivalent to prednisone > 5 mg or equivalent per day for > 3 months

## The DXA Report

The minimum requirements in a standard report include patient demographics, requesting provider, testing indications, instrument manufacturer and model, skeletal sites measured as BMD (g/cm2) for each site, and T-score and Z-score as appropriate [10]. Posteroanterior views of the lumbar spine and hips should be reported in all patients, except in clinical scenarios with lower reliability, such as advanced lumbar degenerative disease, prior spine surgery, or hip arthroplasty. The spine region of interest (ROI) should be reported if vertebrae need to be excluded due to local pathology. If only one vertebra is available for reliable BMD measurement, the lumbar spine should be excluded from making a diagnosis. Z-scores should be reported in premenopausal women and males younger than 50 years of age. Fracture risk prediction tools are commonly reported in the DXA report when there is osteopenia, and fracture risk stratification is beneficial [44]. Errors in the calculated estimation of fracture risk (FRAX score) may occur when patients inaccurately report risk factors, such as misreporting osteoarthritis as rheumatoid arthritis. To maximize clarity for decision-making, ISCD recommends that the DXA report identify the fracture risk calculator used and include the positive risk factors entered into the calculation.

## Unilateral vs. Bilateral DXA Screening

Traditionally, DXA testing measures BMD at two sites, reporting BMD at the lumbar spine and one hip. With the advancement of technology, bilateral hip measurement is now offered and reported by more facilities, with the additional ability to report mean bilateral total hip BMD. The utility and incremental value of bilateral hip reporting are uncertain. The ISCD recommends reporting of bilateral hip BMD [45] as this can be done in a few minutes, it provides low radiation exposure, offers better fracture estimation by capturing the lowest BMD site, and improves the ability for long-term monitoring. Mean bilateral hip BMD uses a larger area than does unilateral measurement and is shown to have superior precision for long-term monitoring [46, 47].

## DXA Monitoring Guidelines

DXA scan can assist in monitoring response to therapy and assist decision-making for consideration of a drug holiday, but indications and timing of repeat BMD measurements remain unclear. Several studies have shown that the rate of decline in BMD varies by individual risk and patient population. BMD remains stable over time in the older community-dwelling population [48, 49], indicating a repeat DXA within 3–7 years may not provide significant additional value. Similarly, a repeat BMD in younger postmenopausal women with low fracture risk based on baseline BMD evaluation, in the absence of clinical risk factors, may not provide significant additional value. Additionally, decreases in BMD may not reliably predict fracture risk and have poor reproducibility, especially in patients on bisphosphonate therapy [50, 51]. Another significant consideration is measurement error, which may lead to lower reliability of serial studies obtained at short intervals [52].

In the 2023 update of Position Statements, ISCD guidelines recommend considering follow-up BMD testing based on clearly defined objectives and when results are likely to influence clinical decision-making [53]. An individualized approach based on risk factors is recommended over systematically repeating the DXA scan at regular intervals [53]. Age and baseline BMD are the strongest factors in determining the interval for follow-up testing. Follow-up testing should be determined on an individualized basis, considering age, baseline BMD, clinical risk factors, and the type of pharmacologic therapy. A shorter interval should be considered in patients at high risk of bone loss, such as patients on medications like glucocorticoids, aromatase inhibitors, or androgen deprivation therapy, patients with inflammatory diseases, or those with premature menopause (< 45 years of age) [11]. Other risk factors for consideration of shorter follow-up intervals include a history of multiple falls and a history of fracture(s). Follow-up BMD should also be considered in the event of the development of new risk factors or new fractures. A significant BMD decrease warrants re-evaluation of fracture risk, consideration for secondary causes of bone loss, and may indicate a need to switch therapy.

## Trabecular Bone Score

Trabecular bone score (TBS) is a computer software program that performs textural analysis of lumbar spine DXA images to provide an indirect measure of bone microarchitecture [54–57], complementing the data from DXA scans, which primarily measure the quantity of bone mineral content.

TBS predicts fracture risk independent of FRAX and clinical risk factors [56, 57], and can enrich fracture risk assessment in conjunction with FRAX or BMD [58]. In particular, TBS is helpful in patients with osteopenia, providing information on (1) risk stratification and (2) decision-making when FRAX is close to intervention thresholds [56]. TBS has been incorporated into the FRAXplus calculator, allowing adjustment of FRAX scores based on TBS [59].

TBS is also helpful for fracture risk assessment in secondary osteoporosis, such as glucocorticoid-induced osteoporosis. Because TBS is not affected by osteophytes, it can be beneficial in assessing fracture risk in patients with spine osteoarthritis [56, 58]. Used with BMD, TBS can be utilized for monitoring treatment response to osteoanabolic agents and long-term denosumab treatment [57, 58].

## Quantitative Computed Tomography

Quantitative computed tomography (QCT) utilizes images from a standard computed tomography scanner to assess BMD [60]. In the spine, QCT can provide a three-dimensional image of bone, thereby providing a volumetric assessment of BMD. QCT in the spine can separate the trabecular bone from the less responsive compact bone [60, 61]. BMD by axial QCT provides volumetric BMD assessment of trabecular bone (measured by grams per cubic centimeter), while a DXA scan only provides an areal BMD assessment (measured in grams per square centimeter) [62]. In the hips, QCT provides areal BMD readings and DXA-equivalent T-scores. Volumetric BMD assessment using QCT methods in the hip, however, is difficult due to the complex structure and variations in BMD in the proximal femur [63]. QCT can also be performed at peripheral sites using smaller, less expensive peripheral CT scanners (pQCT) [60]. High-resolution peripheral quantitative computed tomography (HR-pQCT), with enhanced resolution, provides detailed information about the microarchitecture of peripheral bone. HR-pQCT is only available in research centers and is not yet applicable in general clinical practice. Most guidelines do not clearly define the role of QCT in the diagnosis and management of osteoporosis due to limitations [64], such as a lack of standardization protocols and reference databases for pQCT measurements, and a lack of defined specific treatment thresholds [64].

## Other Uses of DXA

DXA scan is mainly used to assess fracture risk; it can also be used in incomplete atypical femoral fractures (AFFs) [58]. Detection of AFFs via DXA scan is possible by extended-length femur imaging, or full-length femur imaging (FFI) technology, to identify focal periosteal and endosteal thickening in the lateral cortex, as well as transverse lucencies [65, 66]. AFF detection is not widely available in clinical practice, although newer DXA machines offer a software component to assess for AFFs [58].

Whole body DXA scan, an affordable test that can be performed quickly, accurately, and with reproducible results, can also provide an analysis of body composition, including fat, bone mass, and lean tissue. Whole body DXA scan is not used routinely in clinical practice, but is a valuable tool in research settings, such as metabolic syndrome, obesity, and sarcopenia [58, 67].

## Pitfalls in DXA Interpretation

The greatest pitfall of DXA interpretation is relying solely on the cover report of the DXA scan without reviewing the raw images and available data. In the absence of clinical context, DXA reporting and interpretation may be less than accurate, potentially leading to incorrect clinical decision-making. For comparing studies over time to monitor treatment or disease progression, it is crucial to perform DXA scans on the same device at the same facility to allow for a direct comparison.

*Spine positioning*,* Anatomical Variation*,* and Artifacts.*

Spine BMD is measured from L1 to L4, and ideally, the spine is straight with equal soft tissue visible on each side of the vertebrae. When the spine is rotated, the apparent vertebral segment area increases, leading to a low BMD reading, with a neutral effect on bone mineral content [68, 69]. Evaluating the largest area of bone, L1-L4, is ideal. If 1–2 vertebrae are affected by an artifact, such as degenerative changes or scoliosis, those vertebrae should be excluded from consideration. However, when three vertebrae are affected by artifact, the clinical decision should be made based on femoral neck BMD rather than findings in a single vertebra [68]. Correct labeling of L1-L4 is essential. Each labeled vertebra is compared with the normative database to generate a T-score; therefore, mislabeling leads to inaccurate T-scores. Anatomical variations in the lumbar spine can be observed, such as patients having four lumbar vertebrae (7.5% of patients) or six lumbar vertebrae (1.9% of patients). Accurate identification of L1-L4 on the image is crucial, and this can be achieved by counting vertebrae from the sacrum or by identifying them based on their morphology. BMD tends to increase from L1 to L4 in healthy as well as in osteoporotic individuals [70]. Evaluation for artifacts (osteophytes, gallstones, surgical clips) is essential with a sudden change of BMD from one vertebra to the next [68, 71, 72].

## Hip Positioning and Artifacts

For proper hip positioning on the DXA scan, the legs should be abducted and internally rotated by 15–20% [73]. On the DXA image, the femoral shaft should be vertical, with a small area of the lesser trochanter seen on the image [68, 73]. Artifacts potentially interfering with accurate BMD assessment are less common in the hips than in the spine. Common artifacts at the hip include osteoarthritic changes, osteonecrosis, and surgical hardware [74].

## Conclusions, Current State

DXA is a valuable tool in assessing BMD and fracture risk. Combined with FRAX and TBS, DXA provides a comprehensive assessment of fracture risk to guide clinical treatment and monitoring decisions in patients with osteoporosis and low bone mass. In addition to fracture risk estimation, DXA can be used for screening of incomplete AFFs and body mass composition. While it is a valuable tool in assessing fracture risk, it is essential to have familiarity with the treatment guidelines, test utility, limitations, and pitfalls to optimize the interpretation of results and guide clinical decisions for patients with osteoporosis.

## Key References

- LeBoff MS, Greenspan SL, Insogna KL, Lewiecki EM, Saag KG, Singer AJ, Siris ES: **The clinician’s guide to prevention and treatment of osteoporosis**. *Osteoporos Int *2022.
  - This document is an excellent and comprehensive evidence based summary of current osteoporosis screening and treatment recommendations from experts in the field.
- Krueger D, Tanner SB, Szalat A, Malabanan A, Prout T, Lau A, Rosen HN, Shuhart C: **DXA Reporting Updates: 2023 Official Positions of the International Society for Clinical Densitometry**. *J Clin Densitom *2024, **27**:101437.
  - This document provides the latest guidelines on DXA reporting from the ISCD.
- Goel H, Binkley N, Boggild M, Chan WP, Leslie WD, McCloskey E, Morgan SL, Silva BC, Cheung AM: **Clinical Use of Trabecular Bone Score: The 2023 ISCD Official Positions**. *J Clin Densitom *2024,**27**:101452.
  - This document provides the latest guidelines on the utilization of trabecular bone score from the ISCD.
- Force USPST, Nicholson WK, Silverstein M, Wong JB, Chelmow D, Coker TR, Davis EM, Jaen CR, Krousel-Wood M, Lee S, et al.: **Screening for Osteoporosis to Prevent Fractures: US Preventive Services Task Force Recommendation Statement**. *JAMA *2025, **333**:498–508.
  - This document provides the latest osteoporosis screening guidelines from the USPSTF.
- Gani LU, Sritara C, Blank RD, Chen W, Gilmour J, Dhaliwal R, Gill R: **Follow-up Bone Mineral Density Testing: 2023 Official Positions of the International Society for Clinical Densitometry**. *J Clin Densitom *2024, **27**:101440.
  - This document provides the latest guidelines for monitoring BMD with follow up DXA evaluation.
- Humphrey MB, Russell L, Danila MI, Fink HA, Guyatt G, Cannon M, Caplan L, Gore S, Grossman J, Hansen KE, et al.: **2022 American College of Rheumatology Guideline for the Prevention and Treatment of Glucocorticoid-Induced Osteoporosis**. *Arthritis Care Res (Hoboken) *2023, **75**:2405-2419.
  - This document provides the latest practice guidelines by the American College of Rheumatology for osteoporosis screening in patients on long term and/or high dose glucocorticoids.

## Data Availability

No datasets were generated or analysed during the current study.
